# The evaluation of the role of diabetes health literacy and health locus of control on quality of life among type 2 diabetes using the Path analysis

**DOI:** 10.1038/s41598-023-32348-3

**Published:** 2023-04-03

**Authors:** Alireza Jafari, Zohreh Zadehahmad, Vajihe Armanmehr, Mahdi Talebi, Hadi Tehrani

**Affiliations:** 1 Department of Health Education and Health Promotion, School of Health, Social Development and Health Promotion Research Center, Gonabad University of Medical Sciences, Gonabad, Iran; 2 Department of Health Management and Economics, School of Public Health, Tehran University of Medical Sciences, Tehran, Iran; 3 Department of Community and Family Medicine, Faculty of Medicine, Mashhad University of Medical Sciences, Mashhad, Iran; 4 Social Determinants of Health Research Center, Mashhad University of Medical Sciences, Mashhad, Iran; 5 Department of Health Education and Health Promotion, School of Health, Mashhad University of Medical Sciences, Mashhad, Iran; 6 Social Development and Health Promotion Research Center, Gonabad University of Medical Sciences, Gonabad, Iran

**Keywords:** Diseases, Health care

## Abstract

Quality of life (QOL) in patients with diabetes is affected by multiple factors, and this study aimed to determine the effect of health locus of control points (HLOC) and diabetes health literacy (DHL) on QOL in Iranian patients with type 2 diabetes. This cross-sectional study was conducted between October 2021 and February 2022 among 564 people with type 2 diabetes. Patients were selected using proportional stratified sampling and simple random sampling methods. Data were collected using three questionnaires: (1) Multidimensional Health Locus of Control scale (form C), (2) World Health Organization Quality of Life Scale, and (3) Diabetes Health Literacy Scale. Data were analyzed by software’s of SPSS _V22_ and AMOS _V24_. There was a positive and significant correlation between DHL and QOL. There was a positive and significant correlation between the subscales of internal HLOC, and doctors HLOC with QOL. According to the Path analysis results, all variables showed 58.93% of the direct effects and 41.07% of indirect effects of the final model. Numerate health literacy, informational health literacy, communicative health literacy, internal HLOC, other powerful people HLOC, chance HLOC, and doctors HLOC were able to predicted 49% variance of diabetes QOL (R2 = 0.49). The subscales of communicative health literacy, informational health literacy, internal HLOC, doctors HLOC, and chance HLOC had the greatest impact on QOL of people with diabetes. Based on the results of Path analysis, diabetes health literacy and HLOC play an effective role in QOL of diabetic. Therefore, there is a need to design and implement programs to improve the health literacy of patients as well as HLOC to improve QOL of patients.

## Introduction

Type 2 diabetes or diabetes mellitus is a global epidemic^1^. It is one of the metabolic diseases and is a multifactorial disorder characterized by hyperglycemia caused by defects in insulin action, insulin secretion, or both^2^. According to a 2021 study, 536.6 million people worldwide suffer from diabetes, which is expected to increase to 783.2 million by 2045^3^. In Iran, the results of a study showed that 15.14% of the population over the age of 25 suffer from diabetes and the number is expected to increase to 9.2 million by 2030^4^.

Type 2 diabetes reduces people's quality of life (QOL) in a variety of aspects such as social, physical and psychological well -being and increase the economic cost to individuals and society^4,5^. Improving QOL, which refers to the perception of each individual's physical, emotional and social status, is one of the most important goals of a health system^6,7^. The QOL among diabetic is an important parameter for treatment and can affect patients' metabolism^4,8^.

In Iran, the results of a study showed that patients with type 2 diabetes had a moderate QOL^9^. In another study, diabetic had a particularly poor QOL^10^. Type 2 diabetes is a complex and multifaceted nature and is affected by different factors^11,12^. To prevent serious complications and death in people with diabetes, managing diabetes requires self-care behaviors in terms of choosing healthy foods, engaging in physical activity, taking appropriate medications, and controlling blood sugar^13–15^. Practicing self-care behaviors is one of the factors that can help improve QOL of people with diabetes^13,14^.

One of the factors influencing self-care behavior is the health locus of control points (HLOC)^16^. HLOC is a psychological variable can predict diabetic self -management behaviors and including four factors of internal HLOC, doctors HLOC, chance HLOC, and other powerful people HLOC^16,17^. The internal HLOC refer to people's beliefs that they are responsible for their own health^17^. The doctor HLOC refers to people's belief about the role of the physician in their health. People who have a more positive attitude toward the role of the physician in their health are more likely to see your doctor and will further enhance their guidelines^17^. Chance HLOC refer to people's belief that health is affected by luck and fate and person has low control on his or her health^17^. External HLOC (other powerful people) refer to people's belief that health is affected and controlled by other people (such as friends, family members, etc.) and the person does not have much control over his or her health^17^. The results of one study showed that the internal HLOC had the greatest influence on predicting self-care behaviors in patients with type 2 diabetes^18^. There was also a significant relationship between the internal HLOC and regular medicine use among diabetic^19^.

One of the factors affecting the source of health control is health literacy. Health literacy refers to "the extent of people's ability to obtain, process and understand basic health information and access services needed to make appropriate health decisions"^20^. People with diabetes need to become familiar with the scope and complications of the disease in order to manage their condition^21–24^. Studies have shown that health literacy increases health-promoting behaviors^21,22^, reduces disease complications and improves QOL^25,26^.

A number of studies have examined factors that affect QOL in patients with type 2 diabetes^14,26,27^. In some studies, results showed that QOL in people with diabetes can be improved through health literacy and self-efficacy^26–28^. The results of another study showed that the HLOC had a significant impact on QOL of people with diabetes^14^. Searching the data sources, there is no study evaluating the impact of the two variables of diabetes health literacy and HLOC on QOL of patients with type 2 diabetes. In the several studies, only general health literacy in type 2 diabetes was investigated. But in this study, the diabetes health literacy was specifically examined and its impact on HLOC and QOL was examined through Path analysis. Therefore, the aim of this study was to determine the effect HLOC and diabetes health literacy on QOL in Iranian patients with type 2 diabetes.

## Methods

This cross-sectional Path analysis study aimed to investigate the effect of HLOC and diabetes health literacy on QOL in 564 patients with type 2 diabetes between October 2021 and February 2022.

### Sample size

According to the previous study^29^ and the reliability level of 95%, the test capacity of 80%, the similar deviation of the similarity of 0.62 and the accuracy of 0.07, the sample size required was calculated based on the formula below 618. In this study, 54 questionnaires were incomplete, resulting in a response rate of 91%, and finally data from 564 samples were analyzed.

$$n=\frac{{({z}_{1-\frac{\alpha }{2}} +{z}_{1-\beta })}^{2} {(S)}^{2} }{{(d)}^{2}}$$

### Sampling method

In this study, people with type 2 diabetes was entered to study by the proportional stratified sampling. Initially, the number of Torbat Heydariyeh Health Centers and Diabetes Clinics and their populations were determined. Next, the required sample size for each center was chosen by simple random sampling method. In this study, the research objectives were first explained to the participants, and then the consent form was completed by the participants who were satisfied with the study. Then, the questionnaires were completed by self -report and questionnaire of people who were unable to read and write was completed by the questioner. Inclusion criteria for this study were participants who had been diagnosed with diabetes and they had medical records at the health center, participants had diabetes for more than one year, and were satisfied with participating in the study. Questionnaires with incomplete information were excluded during the data analysis phase.

### Data collection instruments

1. ***Demographic questionnaire:*** This part assessed age, sex, age at onset of diabetes, education level, duration of diabetes, occupational status, and marital status.
2. ***Multidimensional Health Locus of Control scale, form C (MHLC-C):*** This scale designed and evaluated in1994 by Wallston. This questionnaire has 18 items and 4 subscales of internal HLOC (6 items), other powerful people HLOC (3 items), chance HLOC (6 items), and doctors HLOC (3 items)^17^. All items are measured on a 6-point Likert scale (completely disagree to completely agree). The validity and reliability of this tool was tested by Mani in an Iranian population^30^ Cronbach's alpha coefficient was reported for all items and subscales of internal, other powerful people, chance, and doctors was reported 0.85, 0.77, 0.64, 0.79 and 0.66, respectively^30^.
3. ***World health organization quality of life scale (WHOQOL):*** This scale has 26 questions and 4 subscales of physical health (7 questions), mental health (6 questions), social relationships (3 questions), environmental health (8 questions), and general QOL and general health (2 questions). Questions are scored between 26 to 130, with higher scores indicating better QOL^31^. The validity and reliability of this questionnaire in Iranian population has been investigated by Nejat^32^ and Cronbach’s alpha for the subscales of physical health, mental health, social relations and environmental health were reported 0.72, 0.70, 0.52, and 0.72, respectively^32^.
4. ***Diabetes health literacy scale (DHL):*** The questionnaire was designed by Lee and consisted of 14 questions and three subscales of Numerate Health Literacy (5 items), Informational Health Literacy (6 items), and Communicative Health Literacy (3 items)^33^. The validity and reliability of this tool in Iranian population has been verified by Moshki^34^ and Cronbach's alpha for all questions and subscales of Numerate Health Literacy, Informational Health Literacy, and Communicative Health Literacy were 0.919, 0.879, 0.865, and 0.784, respectively^34^.

### Statistical analysis

The data in this study were analyzed using SPSS version 22 software. Descriptive statistics of frequencies and percentages were used for qualitative variables, and means and standard deviations were used for quantitative variables. Statistical tests were used, including one-way ANOVA, independent-samples t-test, Pearson correlation, and chi-square test. Independent-samples t-tests were used to compare quantitative and two- categorical qualitative variables. One-way ANOVA was used to compare quantitative variables with three- categorical or more. The Pearson correlation test was used to compare the correlation between two quantitative variables. Chi-square was used to compare two qualitative variables.

### Path analysis

AMOS software version 24 was used to perform path analysis. The Mahalanobis distance statistic is used to find outliers in the data before performing the Path analysis. Additionally, skewness and kurtosis tests were used to check the normality of the data. To evaluate the Path analysis, model fitting indicators such as chi-square ratio to the degree of freedom (× 2/df < 5), comparative fit index (CFI > 0.9), goodness of fit index (GFI > 0.9), incremental fit index (IFI > 0.9), relative fit index (IFI > 0.9), adjusted goodness of fit index (AGFI > 0.8), normed fit index (IFI > 0.9), and root means the square error of approximation (RMSEA ≤ 0.08) were used^35–38^.

### Ethics approval and consent to participate

This study is based on a research project approved by Ethics Committee of Gonabad University of Medical Sciences with the code of ethics IR.MUMS.REC.1401.216. All procedures performed in this study were in accordance with the ethical standards of the institutional and/or national research committee and with the 1964 Helsinki declaration and its later amendments or comparable. Written Informed Consent was obtained from all subjects.

## Results

In this study, the mean (± standard deviation) of patients was 55.81 (± 15.15), the age at onset of diabetes was 46.59 (± 12.43) and the duration of diabetes was 9.4 (± 7.26). According to the results, most participants were female, married, resident in city, had a level of elementary education, and housewives. Most participants reported that they received health information from physicians and health care providers. Additional demographic information is provided in Table 1. The results of Table 2 shows the relationship between demographic variables and DHL. Based on the results of Table 2, there was a significant relationship between sex and DHL and men's DHL were higher than women (p < 0.009).

**Table 1:** Characteristics of demographic variables.

Variables		Data (n = 564)	
		n	%
Sex	Women	363	64.4
	Men	201	35.6
Marital status	Single	11	2
	Married	553	98
Education level	Illiterate	28	5
	Elementary school	202	35.8
	Middle school	89	15.8
	High school	43	7.6
	Diploma	100	17.7
	Associate Degree and bachelor	86	15.2
	Master's degree	16	2.8
Occupation	Housewife	305	54.1
	Employed	94	16.7
	Retired	51	9
	Self-employed	98	17.4
	Unemployed	12	2.1
	Laborer	4	0.7
Inhabitant	Urban	378	67
	Rural	186	33
Have complications	Yes	402	71.3
	No	162	28.7
Income status	< 50 Million Rials (IRR)	32	5.7
	50 -100 Million Rials(IRR)	243	43.1
	> 100 million Rials(IRR)	289	51.2
The age of diabetes begins	≤ 40	191	33.9
	> 40	372	66.1
Diabetes duration	≤ 5	240	42.6
	6–10	122	21.7
	> 10	201	35.7
How do you get more health information?	Physician and health care providers	554	98.2
	Internet, cyberspace	3	0.5
	Newspaper and magazines	1	0.2
	Friends and acquaintances	2	0.4
	Radio, television and satellite	3	0.5
	I do not know	1	0.2

**Table 2:** Relationship between demographic variables and diabetes health literacy (DHL).

Variables		Mean (SD)							
		Informational health literacy	*P*-value	Numerate health literacy	*P*-value	Communicative health literacy	*P*-value	Total DHL	*P*-value
Sex*	Women	20.50(3.72)	0.007^	15.14(3.51)	0.009^	11.45(1.42)	0.246	47.10(8.04)	0.009 ^
	Men	21.40(3.79)		15.95(3.52)		11.59(1.41)		48.95(8.15)	
Marital status*	Single	25.18(2.63)	< 0.001^	19.36(1.02)	< 0.001^	12.81(1.16)	0.002^	57.36(3.61)	< 0.001^
	Married	20.73(3.73)		15.35(3.52)		11.47(1.41)		47.57(8.07)	
Education level**	Illiterate	12.60(2.91)	< 0.001^	9.17(2.73)	< 0.001^	9.00(2.21)	< 0.001^	30.78(6.57)	< 0.001^
	Elementary	18.24(2.35)		13.14(2.87)		10.80(1.27)		42.19(5.58)	
	Middle school	21.22(1.57)		16.07(2.20)		11.75(1.03)		49.05(3.88)	
	high school	22.16(2.08)		15.74(2.54)		12.00(0.57)		49.90(4.63)	
	Diploma	23.36(1.68)		17.47(1.80)		12.18(0.68)		53.01(3.54)	
	Associate Degree and bachelor	24.37(1.68)		18.69(1.23)		12.37(0.88)		55.44(2.86)	
	Master's degree	27(2.33)		20.68(1.77)		13.06(1.23)		60.75(4.75)	
Occupation**	Unemployed	15.75(2.62)	< 0.001^	12.00(3.16)	< 0.001^	9.50(3.69)	< 0.001^	37.25(8.26)	< 0.001^
	Laborer	19.00(2.79)		13.50(3.14)		10.58(0.99)		43.08(6.31)	
	Self-employed	20.18(3.73)		14.86(3.48)		11.23(1.49)		46.28(8.15)	
	Retire	21.84(2.98)		16.00(3.22)		11.96(0.74)		49.80(6.32)	
	Employed	24.30(2.25)		18.45(1.98)		12.34(0.93)		55.10(4.47)	
	Housewife	19.92(3.62)		14.71(3.47)		11.31(1.45)		45.95(7.90)	
Inhabitant *	Urban	21.69(3.56)	< 0.001^	16.22(3.34)	< 0.001^	11.70(1.30)	< 0.001^	49.61(7.64)	< 0.001^
	Rural	19.06(3.57)		13.83(3.37)		11.09(1.55)		44.00(7.77)	
The age of diabetes begins*	≤ 40	22.62(3.23)	< 0.001^	16.89(3.01)	< 0.001^	11.94(1.36)	< 0.001^	51.45(6.92)	< 0.001^
	> 40	19.90(3.70)		14.68(3.55)		11.27(1.40)		45.86(8.06)	
Diabetes duration **	≤ 5	21.62(3.64)	< 0.001^	16.01(3.45)	0.002^	11.75(1.37)	< 0.001^	49.33(7.85)	< 0.001^
	6–10	20.51(3.46)		15.13(3.44)		11.41(1.51)		47.07(8.16)	
	> 10	20.02(3.77)		14.88(3.53)		11.23(1.41)		46.13(8.07)	

* Independents sample T-test, ** One- Way ANOVA, ^ significance level < 0.05.

There was a significant relationship between the level of education and the health literacy of diabetes, and the level of DHL was higher in people with academic education (p < 0.001). There was a significant relationship between job status and DHL, and people with employed job had higher DHL than others (p < 0.001). Also, there was a significant relationship between the residence and DHL and urban people had higher DHL (p < 0.001).

The results in Table 3 shows the relationship between demographic variables and HLOC. According to the results of the Table 3, there was a significant relationship between education level and HLOC, people with high education level think that their disease is less affected by other powerful people HLOC and chance HLOC. They believe that internal HLOC and doctors HLOC were more important in their disease (p < 0.001). There was also a significant relationship between the place of living and the HLOC, and urban people think that their disease is less affected by other powerful people HLOC and chance HLOC. They believe that internal HLOC and doctors HLOC were more important in their disease (p < 0.001). -

**Table 3:** Relationship between demographic variables and health locus of control (HLOC).

Variables		Mean (SD)									
		ChanceHLOC	*P*-value	Other powerful people HLOC	*P*-value	Internal HLOC	*P*-value	Doctors HLOC	*P*-value	Total HLOC	*P*-value
Sex*	Women	12.70 (4.49)	0.064	13.08 (1.86)	0.799	27.66 (3.11)	0.341	15.95 (1.52)	0.468	68.14 (7.59)	0.329
	Men	11.96 (4.31)		13.04 (1.76)		27.92 (3.12)	< 0.001^	16.05 (1.47)		67.49 (7.28)	
Marital status*	Single	8.54 (3.69)	0.003^	10.00 (2.28)	< 0.001^	31.54 (2.80)		16.90 (1.51)	0.041^	67.00 (2.86)	0.684
	Married	12.52 (4.42)		13.13 (1.76)		27.68 (3.08)		15.97 (1.49)		67.92 (7.54)	
Education level**	Illiterate	14.11 (4.77)	< 0.001^	14.83 (3.69)	< 0.001^	25.42 (3.93)	< 0.001^	15.46 (2.87)	< 0.001^	59.50 (14.81)	< 0.001^
	Elementary	14.06 (4.42)		13.90 (1.64)		25.74 (2.19)		15.53 (1.35)		67.31 (8.20)	
	Middle school	12.84 (4.13)		13.15 (1.45)		27.61 (2.61)		15.82 (1.27)		68.26 (7.32)	
	high school	12.79 (4.36)		12.67 (1.58)		28.34 (2.61)		16.04 (1.09)		69.86 (4.40)	
	Diploma	11.89 (4)		12.48 (1.25)		29.32 (2.15)		16.41 (1.19)		69.63 (5.25)	
	Associate Degree and bachelor	9.38 (3.22)		12.06 (1.43)		30.68 (2.39)		16.74 (1.44)		68.88 (3.43)	
	Master's degree	8.25 (2.74)		11.00 (1.75)		30.93 (2.32)		16.81 (1.60)		67.00 (3.59)	
Occupation**	Unemployed	14.33 (7.37)	< 0.001^	16.00 (1.73)	< 0.001^	24.50 (1.73)	< 0.001^	15.25 (1.50)	< 0.001^	62.50 (12.79)	0.443
	Laborer	18.08 (3.96)		12.83 (1.33)		25.83 (3.53)		13.58 (2.23)		70.33 (4.31)	
	Self-employed	12.21 (4.19)		13.50 (1.68)		27.24 (3.09)		15.96 (1.31)		67.62 (6.65)	
	Retire	13.00 (4.29)		13.04 (1.76)		27.92 (2.12)		16.41 (1.20)		67.82 (9.35)	
	Employed	10.00 (3.59)		12.03 (1.58)		30.27 (2.55)		16.65 (1.21)		68.73 (4.79)	
	Housewife	12.96 (4.40)		13.25 (1.83)		27.23 (3.02)		15.82 (1.52)		67.74 (8.06)	
Inhabitant *	Urban	12.09 (4.36)	0.006^	12.92 (1.87)	0.003^	28.31 (3.12)	< 0.001^	16.11 (1.50)	0.007^	69.04 (5.78)	< 0.001^
	Rural	13.24 (4.52)		13.41 (1.67)		26.62 (2.80)		15.75 (1.46)		65.60 (9.70)	
The age of diabetes begins*	≤ 40	11.95 (4.39)	0.069	12.31 (1.63)	< 0.001^	29.05 (2.97)	< 0.001^	16.19 (1.46)	0.021^	68.50 (6.74)	0.168
	> 40	12.68 (4.45)		13.46 (1.79)		27.08 (2.98)		15.88 (1.51)		67.58 (7.82)	
Diabetes duration **	≤ 5	11.77 (4.28)	0.005^	12.77 (1.74)	0.003^	28.17 (3.18)	0.007^	16.17 (1.29)	0.031^	67.35 (7.50)	0.303
	6–10	12.56 (4.51)		13.21 (1.80)		27.77 (2.85)		15.95 (1.38)		68.23 (7.70)	
	> 10	13.1 (4.47)		13.35 (1.87)		27.23 (3.12)		15.98 (1.50)		68.39 (7.32)	

* Independents sample T-test, ** One- Way ANOVA, ^ significance level < 0.05.

Table 4 shows the relationship between demographic variables and QOL. Based on the results, there was a significant relationship between marital status and QOL, with single people having a higher QOL. There was a significant relationship between the education level and QOL, and people with higher education had higher QOL (p = 0.001). There was a significant relationship between job status and QOL, and people with employed job had a higher QOL (p < 0.001). Results also showed that patients with diabetes duration ≤ 5 years had a better QOL (p < 0.001).

**Table 4:** Relationship between demographic variables and quality of life (QOL).

Variables		Mean (SD)											
		Physical health	*P*-value	Mental health	*P*-value	Social	*P*-value	Environmental	*P*-value	Public health	*P*-value	Total QOL	*P*-value
Sex*	Women	24.12 (3.83)	0.634	19.33 (2.99)	0.505	11.06 (1.52)	0.525	26.77 (3.09)	0.538	7.21 (1.07)	0.296	88.50 (11.05)	0.308
	Men	24.29 (4.16)		19.81 (3.26)		11.14 (1.49)		26.96 (3.46)		7.31 (1.11)		89.52 (11.94)	
Marital status*	Single	28.54 (2.11)	< 0.001^	21.36 (2.06)	0.044^	10.18 (2.04)	0.044^	28.00 (2.75)	0.231	7.81 (0.40)	0.001^	95.90 (5.48)	0.001^
	Married	24.09 (3.93)		19.46 (3.10)		11.11 (1.49)		26.82 (3.23)		7.23 (1.09)		88.73 (11.42)	
Education level**	Illiterate	19.89 (3.64)	< 0.001^	17.17 (2.98)	< 0.001^	9.60 (1.66)	< 0.001^	24.46 (3.59)	< 0.001^	6.21 (1.06)	< 0.001^	77.35 (10.19)	< 0.001^
	Elementary	22.20 (3.71)		17.88 (2.74)		10.54 (1.41)		25.25 (3.14)		6.71 (1.19)		82.60 (10.47)	
	Middle school	23.92 (3.69)		19.24 (2.69)		11.24 (1.53)		26.75 (2.34)		7.36 (1.02)		88.52 (9.47)	
	high school	25.46 (3.02)		20.30 (2.66)		11.39 (1.25)		27.58 (2.22)		7.51 (0.82)		92.25 (8.67)	
	Diploma	26.01 (2.85)		20.74 (2.65)		11.61 (1.27)		28.36 (2.81)		7.71 (0.68)		94.43 (8.57)	
	Associate Degree and bachelor	26.97 (2.60)		21.87 (2.27)		11.74 (1.28)		28.66 (2.65)		7.89 (0.50)		97.15 (8.07)	
	Master's degree	28.25 (1.77)		22.81 (1.86)		12.12 (1.40)		30.37 (1.89)		8.06 (0.25)		101.62 (5.36)	
Occupation**	Unemployed	18.50 (3.31)	< 0.001^	16.50 (3.00)	< 0.001^	9.25 (2.50)	< 0.001^	24.75 (6.18)	< 0.001^	6.00 (1.63)	< 0.001^	75.00 (15.03)	< 0.001^
	Laborer	21.08 (5.46)		15.83 (3.80)		9.83 (1.94)		21.83 (2.82)		5.66 (1.49)		74.25 (14.02)	
	Self-employed	23.85 (4.36)		19.56 (3.17)		11.21 (1.30)		26.79 (3.01)		7.27 (1.09)		88.70 (11.33)	
	Retire	24.05 (3.03)		19.86 (2.59)		10.68 (1.27)		28.03 (3.00)		7.31 (0.90)		89.969.25()	
	Employed	26.51 (2.94)		21.64 (2.45)		11.74 (1.33)		28.65 (2.78)		7.82 (0.63)		96.39 (9.05)	
	Housewife	23.79 (3.85)		18.94 (2.93)		10.99 (1.54)		26.32 (3.04)		7.12 (1.10)		87.18 (10.89)	
Inhabitant *	Urban	24.62 (3.76)	< 0.001^	19.91 (3.01)	< 0.001^	11.18 (1.40)	0.050	27.38 (2.91)	< 0.001^	7.38 (1.03)	< 0.001^	90.50 (10.82)	< 0.001^
	Rural	23.27 (4.17)		18.66 (3.10)		10.90 (1.68)		25.74 (3.54)		6.96 (1.14)		85.55 (11.78)	
The age of diabetes begins*	≤ 40	25.76 (3.75)	< 0.001^	20.46 (3.13)	< 0.001^	11.47 (1.60)	< 0.001^	27.60 (3.21)	< 0.001^	7.51 (0.91)	< 0.001^	92.82 (10.99)	< 0.001^
	> 40	23.36 (3.80)		18.99 (2.95)		10.89 (1.42)		26.44 (3.16)		7.11 (1.14)		86.80 (11.03)	
Diabetes duration **	≤ 5	25.23 (3.55)	< 0.001^	20.20 (3.02)	< 0.001^	11.34 (1.36)	< 0.001^	27.36 (3.19)	< 0.001^	7.45 (0.96)	< 0.001^	91.60 (10.55)	< 0.001^
	6–10	24.10 (3.81)		19.32 (2.76)		11.12 (1.65)		26.58 (3.14)		7.19 (1.11)		88.34 (11.19)	
	> 10	22.94 (4.11)		18.74 (3.19)		10.75 (1.50)		26.35 (3.23)		7.01 (1.16)		85.81 (11.35)	

* Independents sample T-test, **One- Way ANOVA, ^ significance level < 0.05.

Table 5 shows the correlation between the variables of the study. Based on the results of Table 5, there was a positive and significant correlation between DHL with internal HLOC (p < 0.001, r = 0.602) and doctors HLOC (p < 0.001, r = 0.342). There was also a negative correlation between DHL with the subscales of other powerful people HLOC (p < 0.001, r = -0.435), and chance HLOC (p < 0.001, r = -0.472). There was a positive and significant correlation between DHL with subscales of physical (p < 0.001, r = 0.585), mental (p < 0.001, r = 0.568), social (p < 0.001, r = 0.456), and environmental (p < 0.001, r = 0.572). There was a positive and significant correlation between DHL and QOL (p < 0.001, r = 0.632). There was a positive and significant correlation between the subscales of internal HLOC (p < 0.001, r = 0.575), and doctors HLOC (p < 0.001, R = 0.428) with QOL. There was also a negative correlation between the other powerful people HLOC (p < 0.001, r = 0.367) and the chance HLOC (p < 0.001, r = -0.443) with QOL (Table 5).

**Table 5:** Pearson correlation between psychological status, DHL, HLOC, and quality of life.

Variables	a	b	c	d	e	f	g	h	i	j	k	l	m	n
Informational	1													
Numerate	0.862*	1												
Communicative	0.722*	0.650*	1											
DHL	0.966*	0.949*	0.793*	1										
Physical	0.578*	0.511*	0.541*	0.585*	1									
Mental	0.560*	0.507*	0.499*	0.568*	0.835*	1								
Social	0.442*	0.417*	0.393*	0.456*	0.613*	0.599*	1							
Environmental	0.551*	0.502*	0.557*	0.572*	0.695*	0.808*	0.531*	1						
Public health	0.516*	0.471*	0.512*	0.534*	0.746*	0.730*	0.568*	0.662*	1					
QOL	0.618*	0.558*	0.583*	0.632*	0.925*	0.941*	0.714*	0.879*	0.817*	1				
Internal HLOC	0.618*	0.528*	0.490*	0.602*	0.563*	0.533*	0.352*	0.491*	0.495*	0.575*	1			
Doctors HLOC	0.337*	0.299*	0.313*	0.342*	0.345*	0.367*	0.354*	0.446*	0.361*	0.428*	0.510*	1		
Chance HLOC	− 0.439*	− 0.458*	− 0.411*	− 0.472*	− 0.365*	− 0.399*	− 0.429*	− 0.406*	− 0.422*	− 0.443*	− 0.428*	− 0.529*	1	
Other powerful people HLOC	− 0.446*	− 0.395*	− 0.338*	− 0.435*	− 0.417*	− 0.325*	− 0.186*	− 0.286*	− 0.319*	− 0.367*	− 0.435*	− 0.078	0.223*	1
Total HLOC	0.072	0.052	-0.071	0.043	0.036	-0.033	0.042	-0.118*	0.006	-0.024	0.254*	0.124*	0.646*	0.318*

* Significance level < 0.001.

**Figure 1:**
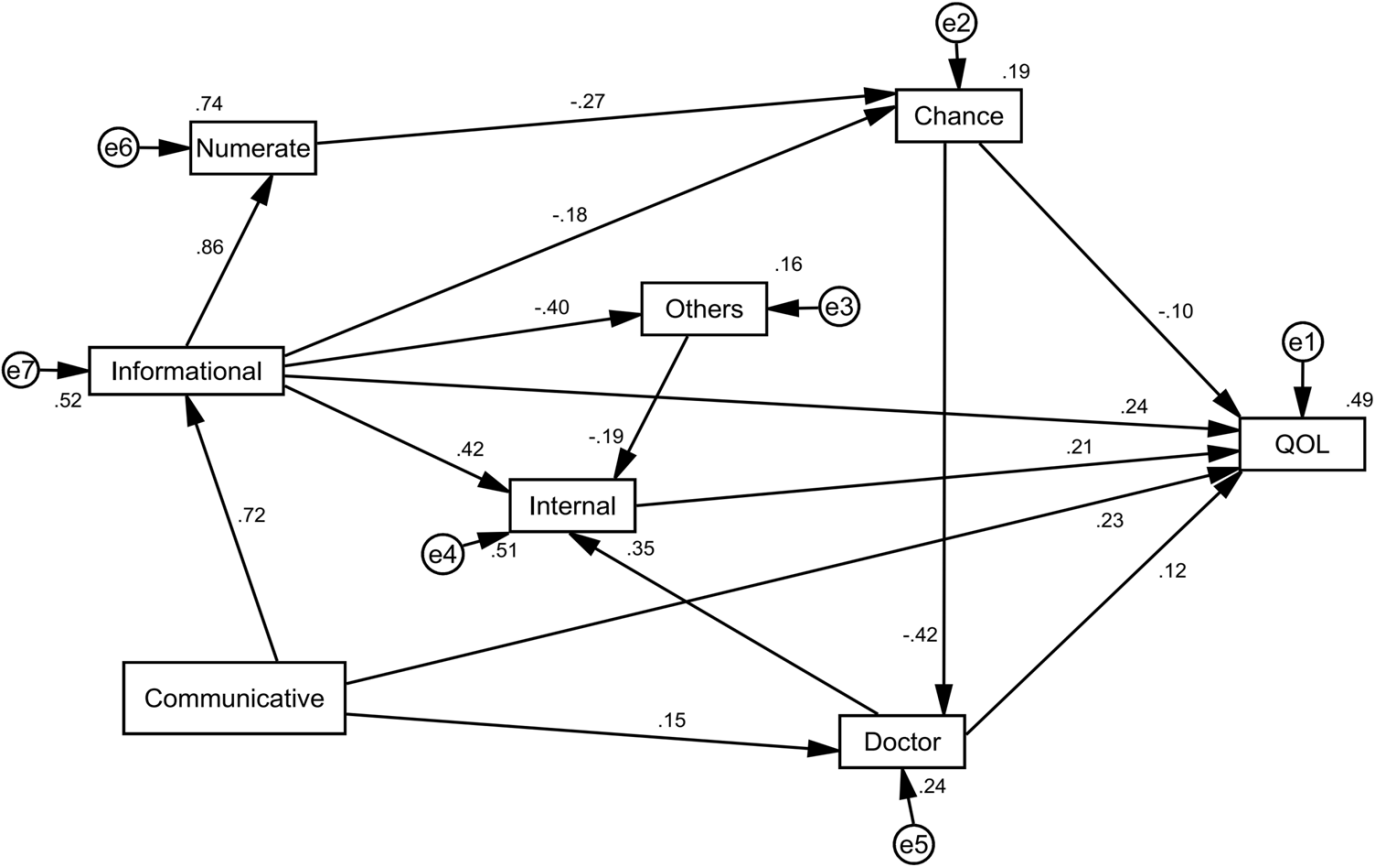
Direct and indirect paths between subscales in Path analysis (R^2^ = 49%). (Diabetes health literacy (DHL): Informational health literacy, Numerate health literacy, Communicative health literacy; Health locus of control (HLOC): Internal HLOC, Doctors HLOC, Chance HLOC, Other powerful people HLOC).

Table 6 shows the model's fitness indicators. Based on the results, the indices had a standard value and final model was acceptable. The index values are shown in Table 6. Results of Table 7 shows the regression coefficient of direct and indirect paths between subscales. Based on the results, all variables showed 58.93% of the direct effects and 41.07% of indirect effects of the final model. Numerate health literacy, information health literacy, communicative health literacy, internal HLOC, other powerful people HLOC, chance HLOC, and doctors HLOC predicted 49% variance of the diabetes QOL (R^2^ = 0.49). The subscales of communicative health literacy (estimate total effect = 0.569), information health literacy (estimate total effect = 0.422), internal HLOC (estimate total effect = 0.214), doctors HLOC (estimate total effect = 0.196), and chance HLOC (estimate total effect = -0.180) had the most impact on the diabetes QOL (Table 7, Fig. 1).

**Table 6:** The model fit indicators.

Goodness of fit indices	Confirmatory factor analysis	Acceptable value
X^2^	31.948	–
df	13	–
X^2^/df	2.485	< 5
P-value	0.002	> 0.05
CFI	0.992	> 0.9
GFI	0.986	> 0.9
IFI	0.992	> 0.9
RFI	0.971	> 0.9
RMSEA	0.051	< 0.08
AGFI	0.961	> 0.8
NFI	0.987	> 0.9

**Table 7:** Direct and indirect paths between subscales in PATH analysis.

Determinants or predictors	Causal effect		
	Direct	Indirect	Total effects
Numerate → Chance	-0.274*	–	− 0.274
Numerate → Doctors	–	0.116	0.116
Numerate → Internal	–	0.041	0.041
Numerate → QOL	–	0.049	0.049
Information → Chance	− 0.181***	-0.236**	− 0.417
Information → Other powerful people	− 0.402*	–	− 0.402
Information → Internal	0.425*	0.139**	0.564
Information → Numerate	0.862*	–	0.862
Information → Doctors	–	0.176**	0.176
Information → QOL	0.240*	0.183**	0.422
Communicative → Information	0.722*	–	0.722
Communicative → QOL	0.234*	0.335**	0.569
Communicative → Doctors	0.151*	0.127**	0.278
Communicative → Numerate	–	0.622**	0.622
Communicative → Chance	–	–0.301**	− 0.301
Communicative → Other powerful people	–	–0.290**	− 0.290
Communicative → Internal	–	0.460**	0.460
Chance → Doctors	− 0.422*		− 0.422
Chance → QOL	− 0.097***	− 0.083**	− 0.180
Chance → Internal	–	− 0.150**	− 0.150
Doctors → Internal	0.354*		0.354
Doctors → QOL	0.120*	0.076**	0.196
Internal → QOL	0.214*	–	0.214
Other powerful people → Internal	-0.191*	–	− 0.191
Other powerful people → QOL	–	− 0.041**	− 0.041
Total causal effect	1.755/2.978	1.223/2.978	2.978
Percantage of direct and indirects effects	58.93%	41.07%	100

*P < 0.001, **P = 0.001, *P < 0.005.

## Discussion

Therefore, the aim of this study was to determine the effect HLOC and diabetes health literacy on QOL in Iranian patients with type 2 diabetes. The results generally showed that there was a relationship between DHL and internal HLOC with QOL. Patients with higher DHL and higher internal HLOC had better QOL. Consistent with the results of this study, Tsai study showed health literacy had a positive correlation with HLOC^39^. Also, The results of Mirzania's study showed that people with higher health literacy had higher internal HLOC and internal HLOC had important role as mediator between health literacy and QOL^40^.

Based on the results of this study, there was a positive and significant correlation between DHL with the internal HLOC and doctors HLOC. There was also a negative correlation between DHL with the other powerful people HLOC and the chance HLOC. In addition, the results of this study showed that DHL with the HLOC had a significant positive correlation with the chances HLOC and external HLOC. people with higher internal HLOC believe that they have the ability to improve their QOL and that their actions control their destiny^41^. People with higher other powerful people HLOC believe that (external control) they are not directly responsible for their own health, thinking that the external HLOC controls their own health, and they cannot play a role in this regard. As a result, they have a sense of disability and inability to control their position, resulting in a reduced QOL^42^. Consistent with the results of this study, results of Son's study showed that health literacy is a predictor of QOL, and adequate health literacy is an important factor in improving patients' QOL^43^.

In this study, health literacy had a positive and significant impact on QOL and subscales of physical, mental, social and environmental health of life. The results of Sun's study showed that health literacy can predicts QOL and adequate health literacy is an important factor in improving QOL in patients^43^. People with low health literacy may pay little attention to their health and thus choose unhealthy behaviors, which reduce their QOL^44^.

Based on the results of this study, numerate health literacy and information health literacy reduces the role of chance and reducing the role of chance enhances patients' QOL. This means that diabetic who have higher information literacy and higher information believe that their disease is not due to the role of other people and chance and consider their role in controlling the disease. This attitude leads people to seek more appropriate self-care behaviors. The results of Mansouri's study showed that self -care behaviors increase in patients with increasing health literacy^45^. Also, in a study aimed at identifying the relationships between health literacy and self -care behaviors, there was a direct and significant relationship between communicative and critical health literacy with self –care behaviors in patients^46^.

Based on the results of Path analysis, communicative health literacy can directly improve QOL of diabetic patients through the influence of doctors' HLOC. This means that patients with higher communicative health literacy are more likely to pay more attention to the role of a physician in their illness and try to manage their illness by regularly visits the doctor and performing the advices provided by the doctor. So, these behaviors help them to enhance their QOL. In general, numerous studies have shown that people with high health literacy can use health services more effectively^47,48^. The results of Cho YI's study showed that health literacy is the most effective and direct way to improve people's health service status^49^.

The path analysis results of this study showed that information health literacy directly reduces the effect of other powerful people HLOC, and enhances the effect of internal HLOC by reducing the effect of other powerful people HLOC. Furthermore, informational health literacy directly increased the effect of internal HLOC and ultimately improved their QOL. This means that the person believes his or her role in managing and caring for their disease is more important. Thus, this attitude can increase their focus on self-care and ultimately improve their QOL. The results of Abredari's study showed that HLOC is associated with self-care behaviors in diabetic and that strengthening internal HLOC improves and enhances self-care behaviors and increases their participation in the treatment process^50^. Baron-Epel study also showed that the internal HLOC is an important mediator between health literacy and the overall health status^51^.

This study had some strengths and limitations. This research was a population-based study, conducted with an appropriate sample of patients, and used validated and reliable instruments that minimized measurement bias for the variables in this study. Due to this research was a cross -sectional study, was that it could only measure relationships between variables. Also, given that the questionnaire was completed based on self-reports, the information may be associated with certain biases.

## Conclusion

Health literacy is a potent factor in HLOC orientation in people with diabetes. Based on the results of Path analysis, DHL and HLOC play an effective role in QOL of type 2 diabetes. Increased DHL and proper HLOC can increase self -care behaviors and these behaviors can help patients' QOL. Therefore, to enhance QOL of patients, it is necessary to design and implement programs to enhance the DHL of patients as well as HLOC to improve QOL of patients. Furthermore, the results of this study suggest the need for more attention to DHL and HLOC belief, especially internal HLOC about diabetes preventive programs.

## Data Availability

All data generated or analysed during this study are included in this published article.

## Abbreviations

MHLC-C: Multidimensional health locus of control scale, form C
HLOC: Health locus of control
DHL: Diabetes health literacy
QOL: Quality of life
